# Supplementary material for: FrCas9 is a CRISPR/Cas9 system with high editing efficiency and fidelity
Source: Nat Commun. 2022 Mar 17;13:1425. doi: 10.1038/s41467-022-29089-8 (PMC8931148; doi:10.1038/s41467-022-29089-8)

Supplementary information

A new CRISPR/Cas9 system with high editing efficiency and fidelity

Cui, et al.

Supplementary Figures 1-9

Supplementary Tables 1-7

Raw gel images in Supplementary information.

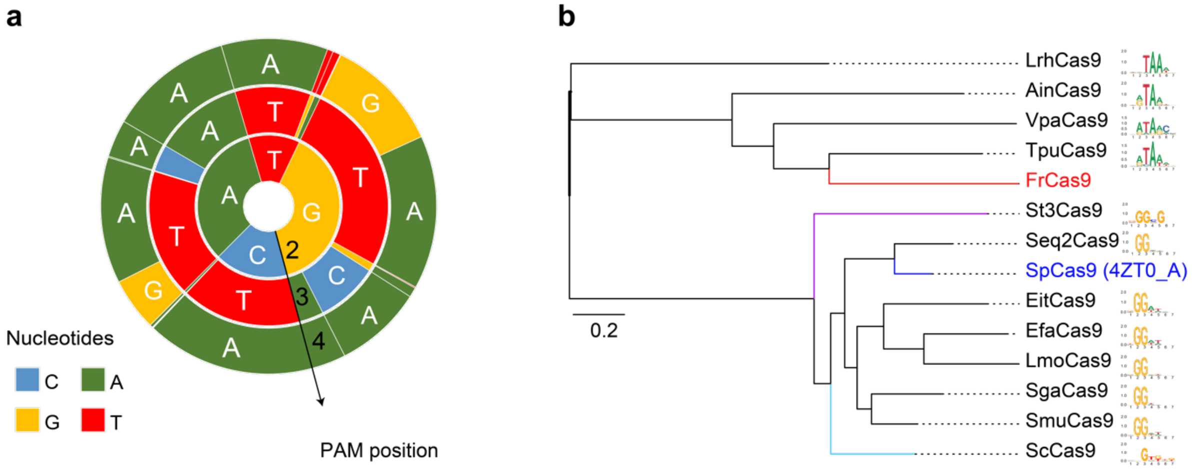

Supplementary Fig. 1 The PAM requirements of FrCas9. a The PAM wheel representation of 2th, 3th and 4th nucleotide positions. **b** The phylogenetic tree of PAM Interacting domains derived from FrCas9 and reported Cas9 orthologs with TA-rich and G-rich PAMs. Source data are provided with this paper.

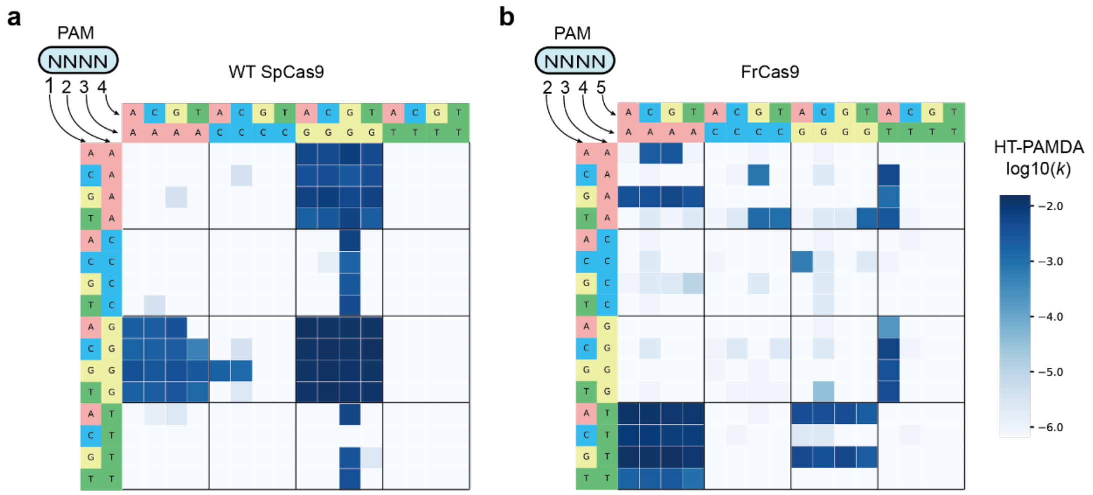

Supplementary Fig. 2 The PAM reference from HT-PAMDA experiments. The representation PAM requirements of WT-SpCas9 **a** and FrCas9 **b** using heatmaps. The heatmap was generated from the same HT-PAMDA data with two replicates per nuclease.

Supplementary Figure 3

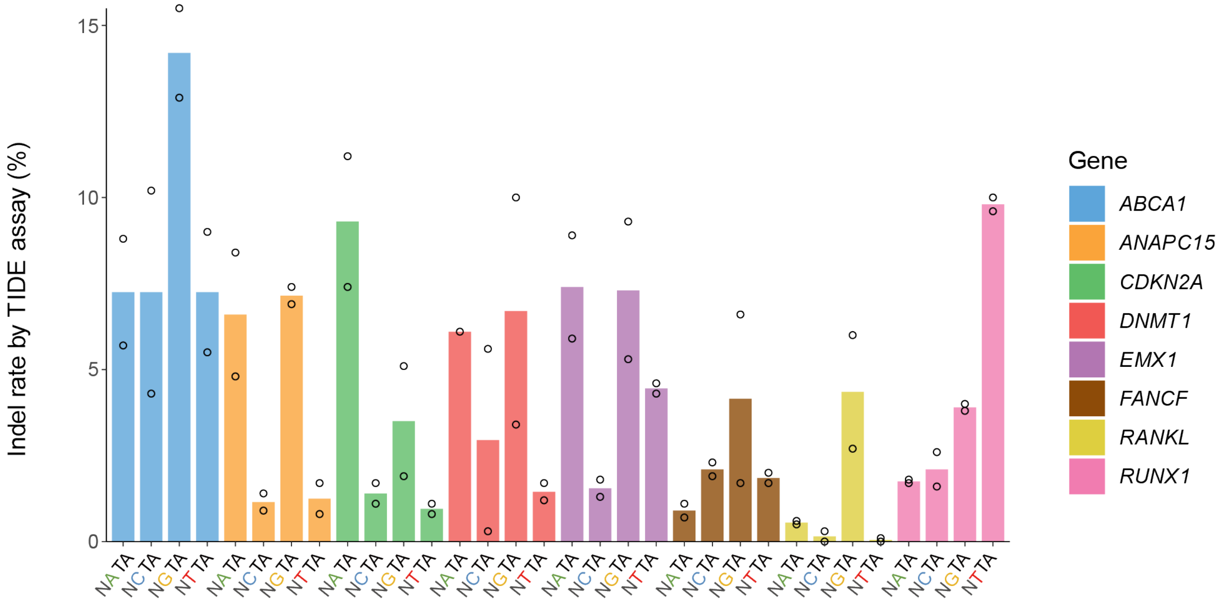

Supplementary Fig. 3 The robust genome editing activity of FrCas9 on 5'-NNTA-3' PAM sequences in human cells. The indel rates induced by FrCas9 in 8. human genes by 32 sgRNAs, which differed in the 2nd PAM base. The assay was generated by TIDE in HEK293T cell line (n = 2). Source data are provided with this paper.

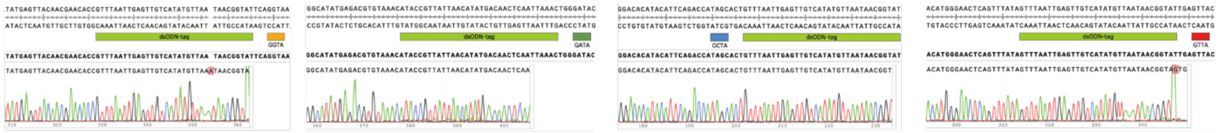

Supplementary Fig. 4 The Sanger sequencing of dsODN breakpoint PCR of *RNF2* gene edited by FrCas9. The dsODN incorporation confirmed that the FrCas9 cleaved targets in the expected positions (3-4 bp upstream the PAM).

Supplementary Figure 5

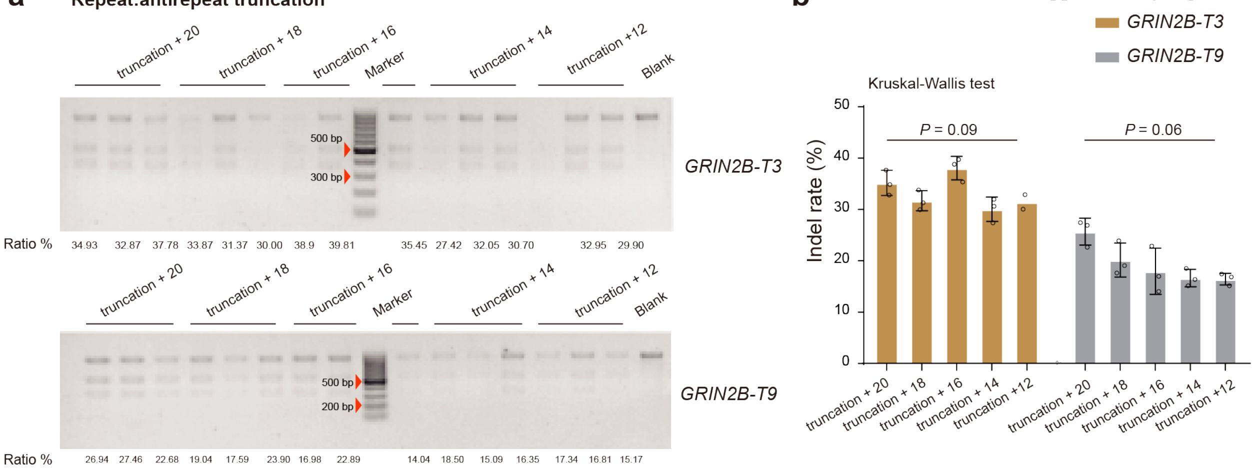

Supplementary Fig. 5 The FrCas9 editing efficiencies with +20 to +12repeat: antirepeat sgRNA truncations. a The gel images of T7E1 assays. Uncropped gel images are provided at the end of Supplementary Information. **b** The statistics of indel rate of different repeat: antirepeat sgRNA truncations at two sites. The data came from the T7E1 assay analyzed by ImageJ. Data are presented as mean +/- S.D. (n=3). Source data are provided with this paper.

Supplementary Figure 6

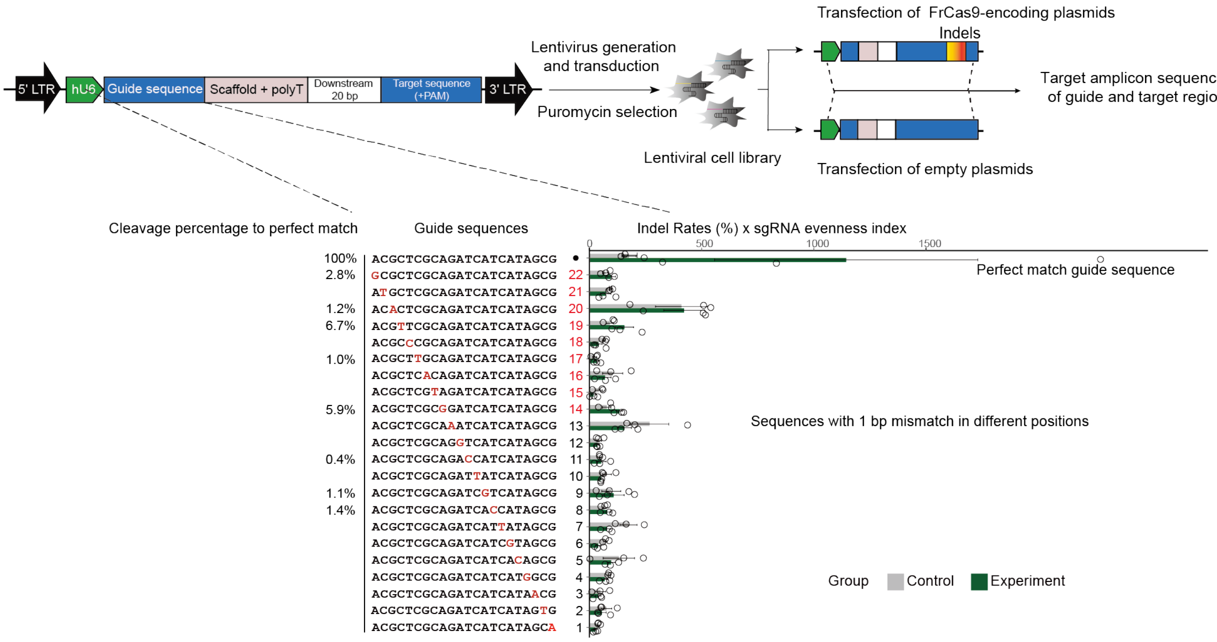

Supplementary Fig. 6 The specificity experiments of length and sequence requirements of FrCas9. The experiments scheme of FrCas9 specificity is shown in above panel. The cleavage efficiencies with sgRNAs of point mutations were assayed by corrected indel frequencies obtained from target amplicon sequencing. A panel of sgRNAs with single nucleotide mismatch (red) were shown below. Data are presented as mean +/- S.E.M. (n=3). Source data are provided with this paper.

Supplementary Figure 7

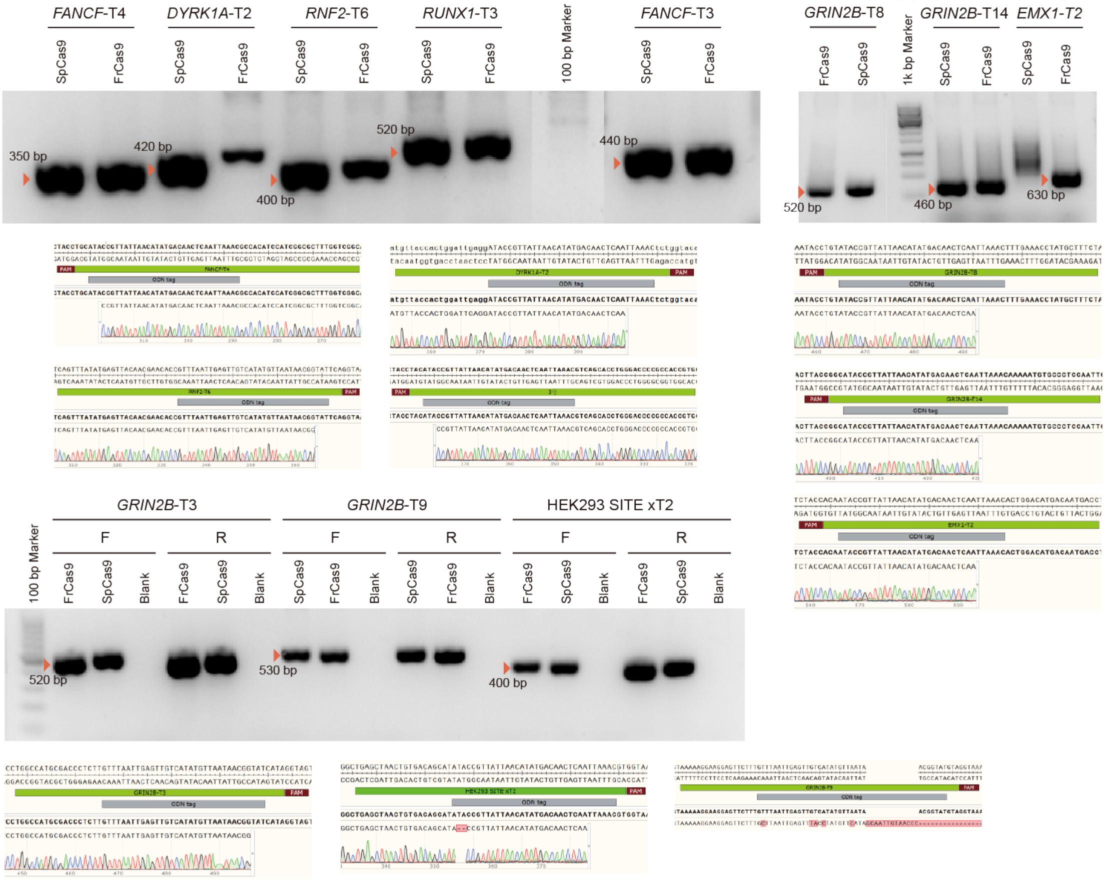

Supplementary Fig. 7 The ODN-breakpoint PCR of 11 sites for GUIDE-seq experiments in HEK293T cell lines. Uncropped gel images are provided at the end of Supplementary information.

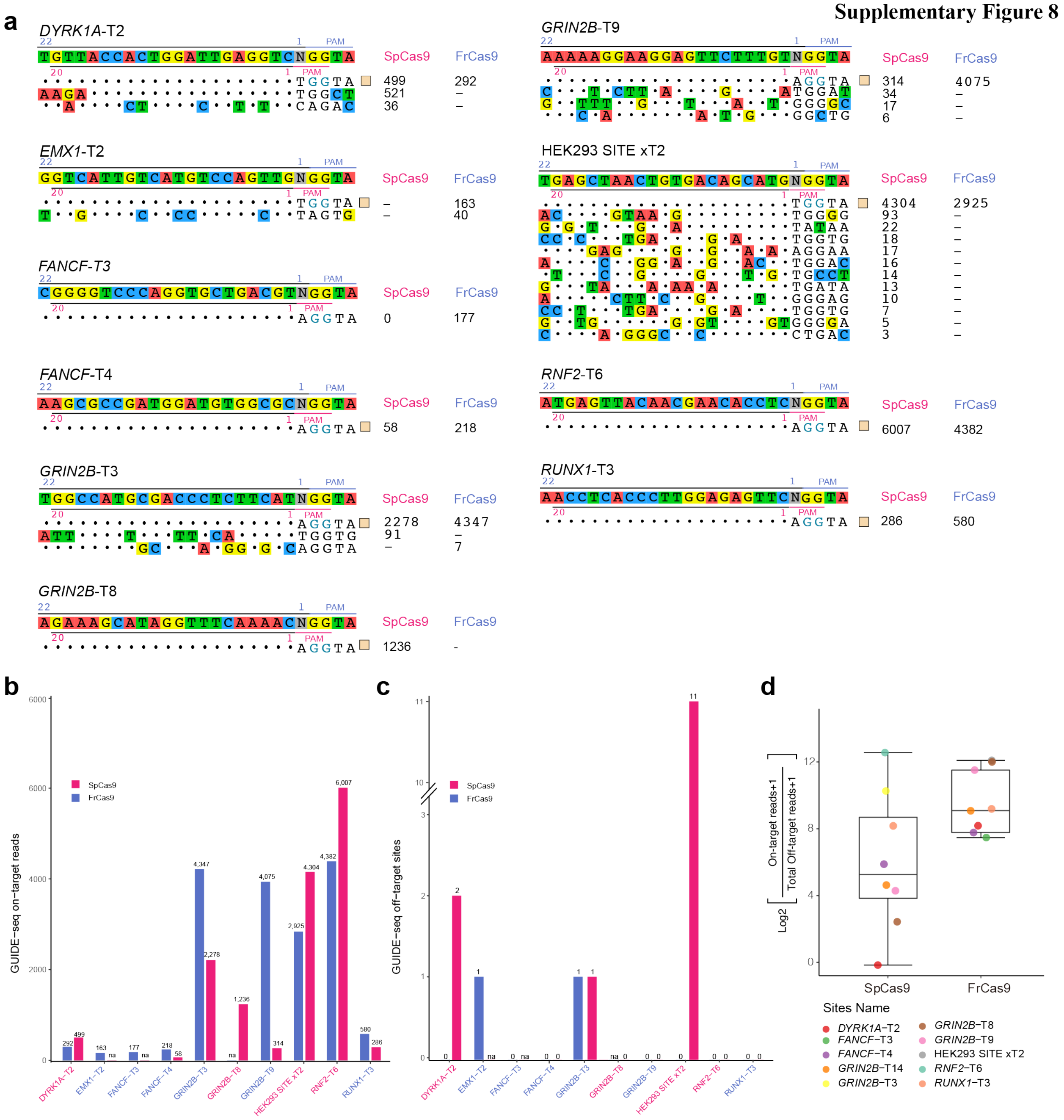

Supplementary Fig. 8 The GUIDE-seq results of 10 sites in U2OS cell line. a The off-targets of SpCas9 and FrCas9 for 10 sites, generated by GUIDE-seq in U2OS cell line. The sgRNA and PAM ranges of SpCas9 (20 nt sgRNA and 3 nt PAM) and FrCas9 (22 nt sgRNA and 4 nt PAM) were marked. The mismatches of either SpCas9 and FrCas9 were highlighted in color and GUIDE-seq read counts of each site were shown on the right side (slash representing no detectable

cleavage). **b** Summary of GUIDE-seq on-target reads of SpCas9 and FrCas9 at above 10 sites. The sites with higher on-target efficiencies by FrCas9 than by SpCas9 were colored in blue, otherwise, colored in red. **c** Summary of off-target counts of SpCas9 and FrCas9 at above 10 sites. **d** The on:off ratio of GUIDE-seq reads of FrCas9 and SpCas9 in U2OS cell line. N=10 sites. Box plots indicate median (middle line), 25th, 75th percentile (box) and 5th and 95th percentile (whiskers). Source data are provided with this paper.

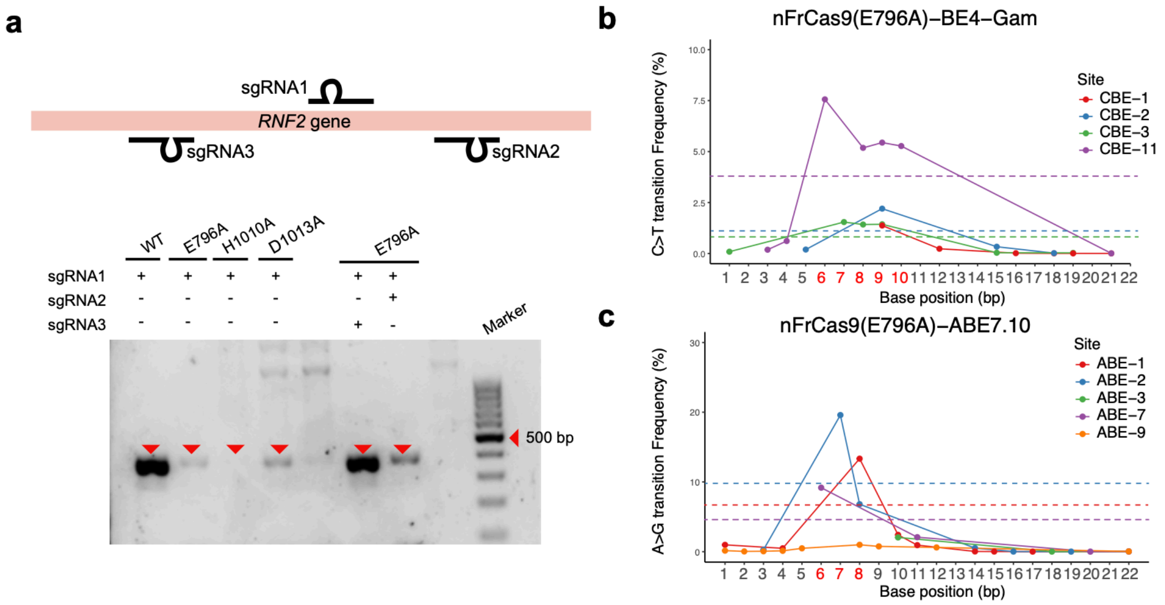

Supplementary Fig. 9 The base editors of FrCas9 and its editing windows. **a** The demonstration of FrCas9 nickases by double-stranded ODN breakpoints PCR. Uncropped gel images are provided at the end of Supplementary information. **b-c** The editing windows of nFrCas9-BE4Gram and nFrCas9-ABE7.10. Source data are provided with this paper.

Supplementary Table 1. The Cas9 protein sequences of the phylogenetic tree.

St1Cas9	MSDLVLGLDIGIGSVGVGILNKVTGEIIHKNSRIFPAAQAENNLVRRTNRQGRRLTRR<br>KKHRRVRLNRLFEESGLITDFTKISINLNPYQLRVKGLTDELSNEELFIALKNMVKHR<br>GISYLDDASDDGNSSIGDYAQIVKENSKQLETKTPGQIQLERYQTYGQLRGDFTVEK<br>DGKKHRLINVFPTSAYRSEALRILQTQQEFNPQITDEFINRYLEILTGKRKYYHGPGNE<br>KSRTDYGRYRTSGETLDNIFGILIGKCTFYPDEFRAAKASYTAQEFNLLNDLNNLTVP<br>TETKKLSKEQKNQIINYVKNEKAMGPAKLFKYIAKLLSCDVADIKGYRIDKSGKAEI<br>HTFEAYRKMKTLETLDIEQMDRETLDKLAYVLTLNTEREGIQEALEHEFADGSFSQK<br>QVDELVQFRKANSSIFGKGWHNFSVKLMMELIPELYETSEEQMTILTRLGKQKTTSS<br>SNKTKYIDEKLLTEEIYNPVVAKSVRQAIKIVNAAIKEYGDFDNIVIEMARETNEDDE<br>KKAIQKIQKANKDEKDAAMLKAANQYNGKAELPHSVFHGHKQLATKIRLWHQQGE<br>RCLYTGKTISIHDLINNSNQFEVDHILPLSITFDDSLANKVLVYATANQEKGQRTPYQ<br>ALDSMDDAWSFRELKAFVRESKTLSNKKKEYLLTEEDISKFDVRKKFIERNLVDTRY<br>ASRVVLNALQEHFRAHKIDTKVSVVRGQFTSQLRRHWGIEKTRDTYHHHAVDALII<br>AASSQLNLWKKQKNTLVSYSEDQLLDIETGELISDDEYKESVFKAPYQHFVDTLKSK<br>EFEDSILFSYQVDSKFNRKISDATIYATRQAKVGKDKADETYVLGKIKDIYTQDGYD<br>AFMKIYKKDKSKFLMYRHDPQTFEKVIEPILENYPNKQINEKGKEVPCNPFLKYKEE<br>HGYIRKYSKKGNGPEIKSLKYYDSKLGNHIDITPKDSNNKVVLQSVSPWRADVYFNK<br>TTGKYEILGLKYADLQFEKGTGTYKISQEKYNDIKKKEGVDSDSEFKFTLYKNDLLL<br>VKDTETKEQQLFRFLSRTMPKQKHYVELKPYDKQKFEGGEALIKVLGNVANSGQCK<br>KGLGKSNISIYKVRTDVLGNQHIIKNEGDKPKLDF
St3C	MTKPYSIGLDIGTNSVGWAVITDNYKVPSKKMKVLGNTSKKYIKKNLLGVLLFDSGI<br>TAEGRRLKRTARRRYTRRRNRILYLQEIFSTEMATLDDAFFQRLDDSFLVPDDKRDS

as<br>9	KYPIFGNLVEEKVYHDEFPTIYHLRKYLADSTKKADLRLVYLALAHMIKYRGHFLIE<br>GEFNSKNNDIQKNFQDFLDTYNAIFESDLSLENSKQLEEIVKDKISKLEKKDRILKLFP<br>GEKNSGIFSEFLKLIVGNQADFRKCFNLDEKASLHFSKESYDEDLETLLGYIGDDYSD<br>VFLKAKKLYDAILLSGFLTVTDNETEAPLSSAMIKRYNEHKEDLALLKEYIRNISLKT<br>YNEVFKDDTKNGYAGYIDGKTNQEDFYVYLKNLLAEFEGADYFLEKIDREDFLRKQ<br>RTFDNGSIPYQIHLQEMRAILDKQAKFYPFLAKNKERIEKILTFRIPYYVGPLARGNSD<br>FAWSIRKRNEKITPWNFEDVIDKESSAEAFINRMTSFDLYLPEEKVLPKHSLLYETFN<br>VYNELTKVRFIAESMRDYQFLDSKQKKDIVRLYFKDKRKVTDKDIIEYLHAIYGYDG<br>IELKGIEKQFNSSLSTYHDLLNIINDKEFLDDSSNEAIIEEIIHTLTIFEDREMIKQRLSKF<br>ENIFDKSVLKKLSRRHYTGWGKLSAKLINGIRDEKSGNTILDYLIDDGISNRNFMQLI<br>HDDALSFKKKIQKAQIIGDEDKGNIKEVVKSLPGSPAIKKGILQSIKIVDELVKVMGG<br>RKPESIVVEMARENQYTNQGKSNSQQRLKRLEKSLKELGSKILKENIPAKLSKIDNNA<br>LQNDRLYLYYLQNGKDMYTGDDLDIDRLSNYDIDHIIPQAFLKDNSIDNKVLVSSAS<br>NRGKSDDFPSLEVVKKRKTFWYQLLKSKLISQRKFDNLTKAERGGLLPEDKAGFIQR<br>QLVETRQITKHVARLLDEKFNNKKDENNRAVRTVKIITLKSTLVSQFRKDFELYKVR<br>EINDFHHAHDAYLNAVIASALLKKYPKLEPEFVYGDYPKYNSFRERKSATEKVYFYS<br>NIMNIFKKSISLADGRVIERPLIEVNEETGESVWNKESDLATVRRVLSYPQVNVVKKV<br>EEQNHGLDRGKPKGLFNANLSSKPKPNSNENLVGAKEYLDPKKYGGYAGISNSFAV<br>LVKGTIEKGAKKKITNVLEFQGISILDRINYRKDKLNFLLEKGYKDIELIIELPKYSLFE<br>LSDGSRRMLASILSTNNKRGEIHKGNQIFLSQKFVKLLYHAKRISNTINENHRKYVEN<br>HKKEFEELFYYILEFNENYVGAKKNGKLLNSAFQSWQNHSIDELCSSFIGPTGSERKG<br>LFELTSRGSAADFEFLGVKIPRYRDYTPSSLLKDATLIHQSVTGLYETRIDLAKLGEG

ScCas9	MEKKYSIGLDIGTNSVGWAVITDDYKVPSKKFKVLGNTNRKSIKKNLMGALLFDSG<br>ETAEATRLKRTARRRYTRRKNRIRYLQEIFANEMAKLDDSFFQRLEESFLVEEDKKN<br>ERHPIFGNLADEVAYHRNYPTIYHLRKKLADSPEKADLRLIYLALAHIIKFRGHFLIEG<br>KLNAENSDVAKLFYQLIQTYNQLFEESPLDEIEVDAKGILSARLSKSKRLEKLIAVFPN<br>EKKNGLFGNIIALALGLTPNFKSNFDLTEDAKLQLSKDTYDDDLDELLGQIGDQYAD<br>LFSAAKNLSDAILLSDILRSNSEVTKAPLSASMVKRYDEHHQDLALLKTLVRQQFPE<br>KYAEIFKDDTKNGYAGYVGIGIKHRKRTTKLATQEEFYKFIKPILEKMDGAEELLAK<br>LNRDDLLRKQRTFDNGSIPHQIHLKELHAILRRQEEFYPFLKENREKIEKILTFRIPYYV<br>GPLARGNSRFAWLTRKSEEAITPWNFEEVVDKGASAQSFIERMTNFDEQLPNKKVLP<br>KHSLLYEYFTVYNELTKVKYVTERMRKPEFLSGEQKKAIVDLLFKTNRKVTVKQLK<br>EDYFKKIECFDSVEIIGVEDRFNASLGTYHDLLKIIKDKDFLDNEENEDILEDIVLTLTL<br>FEDREMIEERLKTYAHLFDDKVMKQLKRRHYTGWGRLSRKMINGIRDKQSGKTILD<br>FLKSDGFSNRNFMQLIHDDSLTFKEEIEKAQVSGQGDSLHEQIADLAGSPAIKKGILQ<br>TVKIVDELVKVMGHKPENIVIEMARENQTTTKGLQQSRERKKRIEEGIKELESQILKE<br>NPVENTQLQNEKLYLYYLQNGRDMYVDQELDINRLSDYDVDHIVPQSFIKDDSIDN<br>KVLTRSVENRGKSDNVPSEEVVKKMKNYWRQLLNAKLITQRKFDNLTKAERGGLS<br>EADKAGFIKRQLVETRQITKHVARILDSRMNTKRDKNDKPIREVKVITLKSKLVSDFR<br>KDFQLYKVRDINNYHHAHDAYLNAVVGTALIKKYPKLESEFVYGDYKVYDVRKMI<br>AKSEQEIGKATAKRFFYSNIMNFFKTEVKLANGEIRKRPLIETNGETGEVVWNKEKD<br>FATVRKVLAMPQVNIVKKTEVQTGGFSKESILSKRESAKLIPRKKGWDTRKYGGFGS<br>PTVAYSILVVAKVEKGKAKKLKSVKVLVGITIMEKGSYEKDPIGFLEAKGYKDIKKE<br>LIFKLPKYSLFELENGRRRMLASATELQKANELVLPQHLVRLLYYTQNISATTGSNNL

	GYIEQHREEFKEIFEKIIDFSEKYILKNKVNSNLKSSFDEQFAVSDSILLSNSFVSLLKY<br>TSFGASGGFTFLDLDVKQGRLRYQTVTEVLDATLIYQSITGLYETRTDLSQLGGD
SauriCas9	MQENQQKQNYILGLDIGITSVGYGLIDSKTREVIDAGVRLFPEADSENNSNRRSKRG<br>ARRLKRRRIHRLNRVKDLLADYQMIDLNNVPKSTDPYTIRVKGLREPLTKEEFAIALL<br>HIAKRRGLHNISVSMGDEEQDNELSTKQQLQKNAQQLQDKYVCELQLERLTNINKV<br>RGEKNRFKTEDFVKEVKQLCETQRQYHNIDDQFIQQYIDLVSTRREYFEGPGNGSPY<br>GWDGDLLKWYEKLMGRCTYFPEELRSVKYAYSADLFNALNDLNNLVVTRDDNPK<br>LEYYEKYHIIENVFKQKKNPTLKQIAKEIGVQDYDIRGYRITKSGKPQFTSFKLYHDL<br>KNIFEQAKYLEDVEMLDEIAKILTIYQDEISIKKALDQLPELLTESEKSQIAQLTGYTG<br>THRLSLKCIHIVIDELWESPENQMEIFTRLNLKPKKVEMSEIDSIPTTLVDEFILSPVVK<br>RAFIQSIKVINAVINRFGLPEDIIIELAREKNSKDRRKFINKLQKQNEATRKKIEQLLAK<br>YGNTNAKYMIEKIKLHDMQEGKCLYSLEAIPLEDLLSNPTHYEVDHIIPRSVSFDNSL<br>NNKVLVKQSENSKKGNRTPYQYLSSNESKISYNQFKQHILNLSKAKDRISKKKRDML<br>LEERDINKFEVQKEFINRNLVDTRYATRELSNLLKTYFSTHDYAVKVKTINGGFTNH<br>LRKVWDFKKHRNHGYKHHAEDALVIANADFLFKTHKALRRTDKILEQPGLEVNDT<br>TVKVDTEEKYQELFETPKQVKNIKQFRDFKYSHRVDKKPNRQLINDTLYSTREIDGE<br>TYVVQTLKDLYAKDNEKVKKLFTERPQKILMYQHDPKTFEKLMTILNQYAEAKNPL<br>AAYYEDKGEYVTKYAKKGNGPAIHKIKYIDKKLGSYLDVSNKYPETQNKLVKLSLK<br>SFRFDIYKCEQGYKMVSIGYLDVLKKDNYYYIPKDKYEAEKQKKKIKESDLFVGSFY<br>YNDLIMYEDELFRVIGVNSDINNLVELNMVDITYKDFCEVNNVTGEKRIKKTIGKRV<br>VLIEKYTTDILGNLYKTPLPKKPQLIFKRGEL

**SaCas9**	MKRNYILGLDIGITSVGYGIIDYETRDVIDAGVRLFKEANVENNEGRRSKRGARRLK<br>RRRRHRIQRVKKLLFDYNLLTDHSELSGINPYEARVKGLSQKLSEEEFSAALLHLAK<br>RRGVHNVNEVEEDTGNELSTKEQISRNSKALEEKYVAELQLERLKKDGEVRGSINRF<br>KTSDYVKEAKQLLKVQKAYHQLDQSFIDTYIDLLETRRTYYEGPGEGSPFGWKDIKE<br>WYEMLMGHCTYFPEELRSVKYAYNADLYNALNDLNNLVITRDENEKLEYYEKFQII<br>ENVFKQKKKPTLKQIAKEILVNEEDIKGYRVTSTGKPEFTNLKVYHDIKDITARKEIIE<br>NAELLDQIAKILTIYQSSEDIQEELTNLNSELTQEEIEQISNLKGYTGTHNLSLKAINLIL<br>DELWHTNDNQIAIFNRLKLVPKKVDLSQQKEIPTTLVDDFILSPVVKRSFIQSIKVINAI<br>IKKYGLPNDIIIELAREKNSKDAQKMINEMQKRNRQTNERIEEIIRTTGKENAKYLIEK<br>IKLHDMQEGKCLYSLEAIPLEDLLNNPFNYEVDHIIPRSVSFDNSFNNKVLVKQEENS<br>KKGNRTPFQYLSSSDSKISYETFKKHILNLAKGKGRISKTKKEYLLEERDINRFSVQK<br>DFINRNLVDTRYATRGLMNLLRSYFRVNNLDVKVKSINGGFTSFLRRKWKFKKERN<br>KGYKHHAEDALIIANADFIFKEWKKLDKAKKVMENQMFEEKQAESMPEIETEQEYK<br>EIFITPHQIKHIKDFKDYKYSHRVDKKPNRELINDTLYSTRKDDKGNTLIVNNLNGLY<br>DKDNDKLKKLINKSPEKLLMYHHDPQTYQKLKLIMEQYGDEKNPLYKYYEETGNY<br>LTKYSKKDNGPVIKKIKYYGNKLNAHLDITDDYPNSRNKVVKLSLKPYRFDVYLDN<br>GVYKFVTVKNLDVIKKENYYEVNSKCYEEAKKLKKISNQAEFIASFYNNDLIKINGE<br>LYRVIGVNNDLLNRIEVNMIDITYREYLENMNDKRPPRIIKTIASKTQSIKKYSTDILG<br>NLYEVKSKKHPQIIKKG
NmCas9	MAAFKPNSINYILGLDIGIASVGWAMVEIDEEENPIRLIDLGVRVFERAEVPKTGDSL<br>AMARRLARSVRRLTRRRAHRLLRTRRLLKREGVLQAANFDENGLIKSLPNTPWQLR<br>AAALDRKLTPLEWSAVLLHLIKHRGYLSQRKNEGETADKELGALLKGVAGNAHAL<br>QTGDFRTPAELALNKFEKESGHIRNQRSDYSHTFSRKDLQAELILLFEKQKEFGNPHV

	SGGLKEGIETLLMTQRPALSGDAVQKMLGHCTFEPAEPKAAKNTYTAERFIWLTKL<br>NNLRILEQGSERPLTDTERATLMDEPYRKSKLTYAQARKLLGLEDTAFFKGLRYGKD<br>NAEASTLMEMKAYHAISRALEKEGLKDKKSPLNLSPELQDEIGTAFSLFKTDEDITGR<br>LKDRIQPEILEALLKHISFDKFVQISLKALRRIVPLMEQGKRYDEACAEIYGDHYGKK<br>NTEEKIYLPPIPADEIRNPVVLRALSQARKVINGVVRRYGSPARIHIETAREVGKSFKD<br>RKEIEKRQEENRKDREKAAAKFREYFPNFVGEPKSKDILKLRLYEQQHGKCLYSGKE<br>INLGRLNEKGYVEIDHALPFSRTWDDSFNNKVLVLGSENQNKGNQTPYEYFNGKDN<br>SREWQEFKARVETSRFPRSKKQRILLQKFDEDGFKERNLNDTRYVNRFLCQFVADR<br>MRLTGKGKKRVFASNGQITNLLRGFWGLRKVRAENDRHHALDAVVVACSTVAMQ<br>QKITRFVRYKEMNAFDGKTIDKETGEVLHQKTHFPQPWEFFAQEVMIRVFGKPDGK<br>PEFEEADTLEKLRTLLAEKLSSRPEAVHEYVTPLFVSRAPNRKMSGQGHMETVKSAK<br>RLDEGVSVLRVPLTQLKLKDLEKMVNREREPKLYEALKARLEAHKDDPAKAFAEPF<br>YKYDKAGNRTQQVKAVRVEQVQKTGVWVRNHNGIADNATMVRVDVFEKGDKYY<br>LVPIYSWQVAKGILPDRAVVQGKDEEDWQLIDDSFNFKFSLHPNDLVEVITKKARMF<br>GYFASCHRGTGNINIRIHDLDHKIGKNGILEGIGVKTALSFQKYQIDELGKEIRPCRLK<br>KRPPVR
CjCas9	MARILAFDIGISSIGWAFSENDELKDCGVRIFTKVENPKTGESLALPRRLARSARKRLARRKA<br>RLNHLKHLIANEFKLNYEDYQSFDESLAKAYKGSLISPYELRFRALNELLSKQDFARVILHIA<br>KRRGYDDIKNSDDKEKGAILKAIKQNEEKLANYQSVGEYLYKEYFQKFKENSKEFTNVRNK<br>KESYERCIAQSFLKDELKLIFKKQREFGFSFSKKFEEEVLSVAFYKRALKDFSHLVGNCSFFTD<br>EKRAPKNSPLAFMFVALTRIINLLNNLKNTEGILYTKDDLNALLNEVLKNGTLTYKQTKKLL<br>GLSDDYEFKGEKGTYFIEFKKYKEFIKALGEHNLSQDDLNEIAKDITLIKDEIKLKKALAKYD<br>LNQNQIDSLSKLEFKDHLNISFKALKLVTPLMLEGKKYDEACNELNLKVAINEDKKDFLPAF

	NETYYKDEVTNPVVLRAIKEYRKVLNALLKKYGKVHKINIELAREVGKNHSQRAKIEKEQNENYKAKKDAELECEKLGLKINSKNILKLRLFKEQKEFCAYSGEKIKISDLQDEKMLEIDHIYPYSRSFDDSYMNKVLVFTKQNQEKLNQTPFEAFGNDSAKWQKIEVLAKNLPTKKQKRILDKNYKDKEQKNFKDRNLNDTRYIARLVLNYTKDYLDFLPLSDDENTKLNDTQKGSKVHVEAKSGMLTSALRHTWGFSAKDRNNHLHHAIDAVIIAYANNSIVKAFSDFKKEQESNSAELYAKKISELDYKNKRKFFEPFSGFRQKVLDKIDEIFVSKPERKKPSGALHEETFRKEEEFYQSYGGKEGVLKALELGKIRKVNGKIVKNGDMFRVDIFKHKKTNKFYAVPIYTMDFALKVLPNKAVARSKKGEIKDWILMDENYEFCFSLYKDSLILIQTKDMQEPEFVYYNAFTSSTVSLIVSKHDNKFETLSKNQKILFKNANEKEVIAKSIGIQNLKVFEKYIVSALGEVTKAEFRQREDFKK
CcCas9	MKYTLGLDVGIASVGWAVIDKDNNKIIDLGVRCFDKAEESKTGESLATARRIARGMRRRISRRSQRLRLVKKLFVQYEIIKDSSEFNRIFDTSRDGWKDPWELRYNALSRILKPYELVQVLTHITKRRGFKSNRKEDLSTTKEGVVITSIKNNSEMLRTKNYRTIGEMIFMETPENSNKRNKVDEYIHTIAREDLLNEIKYIFSIQRKLGSPFVTEKLEHDFLNIWEFQRPFASGDSILSKVGKCTLLKEELRAPTSCYTSEYFGLLQSINNLVLVEDNNTLTLNNDQRAKIIEYAHFKNEIKYSEIRKLLDIEPEILFKAHNLTHKNPSGNNESKKFYEMKSYHKLKSTLPTDIWGKLHSNKESLDNLFYCLTVYKNDNEIKDYLQANNLDYLIEYIAKLPTFNKFKHLSLVAMKRIIPFMEKGYKYSDACNMAELDFTGSSKLEKCNKLTVEPIIENVTNPVVIRALTQARKVINAIIQKYGLPYMVNIELAREAGMTRQDRDNLKKEHENNRKAREKISDLIRQNGRVASGLDILKWRLWEDQGGRCAYSGKPIPVCDLLNDSLTQIDHIYPYSRSMDDSYMNKVLVLTDENQNKRSYTPYEVWGSTEKWEDFEARIYSMHLPQSKEKRLLNRNFITKDLDSFISRNLNDTRYISRFLKNYIESYLQFSNDSPKSCVVCVNGQCTAQLRSRWGLNKNREESDLHHALDAAVIACADRKIIKEITNYYNERENHNYKVKYPLPWHSFRQDLMETLAGVFISRAPRRKITGPAHDETIRSPKHFNKGLTSVKIPLTTVTLEKLETMVKNTKGGISDKAVYNVLKNRLIEHNNKPLKAFAEKIYKPLKNGTNGAIIRSIRVETPSYTGVFRNEGKGISDNSLMVRVDVFKKKDKYYLVPIYVAHMIKKELPSKAIVPLKPESQWELIDSTHEFLFSLYQN

	DYLVIKTKKGITEGYYRSCHRGTGSLSLMPHFANNKNVKIDIGVRTAISIEKYNVDILGNKSIV<br>KGEPRRGMEKYNSFKSN
**AceCas9**	MGGSEVGTVPVTWRLGVDVGERSIGLAAVSYEEDKPKEILAAVSWIHDGGVGDERSGASRL<br>ALRGMARRARRLRRFRRARLRDLDMLLSELGWTPLPDKNVSPVDAWLARKRLAEEYVVDE<br>TERRRLLGYAVSHMARHRGWRNPWTTIKDLKNLPQPSDSWERTRESLEARYSVSLEPGTVG<br>QWAGYLLQRAPGIRLNPTQQSAGRRAELSNATAFETRLRQEDVLWELRCIADVQGLPEDVV<br>SNVIDAVFCQKRPSVPAERIGRDPLDPSQLRASRACLEFQEYRIVAAVANLRIRDGSGSRPLSL<br>EERNAVIEALLAQTERSLTWSDIALEILKLPNESDLTSVPEEDGPSSLAYSQFAPFDETSARIAE<br>FIAKNRRKIPTFAQWWQEQDRTSRSDLVAALADNSIAGEEEQELLVHLPDAELEALEGLALP<br>SGRVAYSRLTLSGLTRVMRDDGVDVHNARKTCFGVDDNWRPPLPALHEATGHPVVDRNLA<br>ILRKFLSSATMRWGPPQSIVVELARGASESRERQAEEEAARRAHRKANDRIRAELRASGLSDP<br>SPADLVRARLLELYDCHCMYCGAPISWENSELDHIVPRTDGGSNRHENLAITCGACNKEKGR<br>RPFASWAETSNRVQLRDVIDRVQKLKYSGNMYWTRDEFSRYKKSVVARLKRRTSDPEVIQSI<br>ESTGYAAVALRDRLLSYGEKNGVAQVAVFRGGVTAEARRWLDISIERLFSRVAIFAQSTSTK<br>RLDRRHHAVDAVVLTTLTPGVAKTLADARSRRVSAEFWRRPSDVNRHSTEEPQSPAYRQW<br>KESCSGLGDLLISTAARDSIAVAAPLRLRPTGALHEETLRAFSEHTVGAAWKGAELRRIVEPE<br>VYAAFLALTDPGGRFLKVSPSEDVLPADENRHIVLSDRVLGPRDRVKLFPDDRGSIRVRGGA<br>AYIASFHHARVFRWGSSHSPSFALLRVSLADLAVAGLLRDGVDVFTAELPPWTPAWRYASIA<br>LVKAVESGDAKQVGWLVPGDELDFGPEGVTTAAGDLSMFLKYFPERHWVVTGFEDDKRIN<br>LKPAFLSAEQAEVLRTERSDRPDTLTEAGEILAQFFPRCWRATVAKVLCHPGLTVIRRTALGQ<br>PRWRRGHLPYSWRPWSADPWSGGTP*
**Nme2C**	MAAFKPNPINYILGLDIGIASVGWAMVEIDEEENPIRLIDLGVRVFERAEVPKTGDSLAMARR<br>LARSVRRLTRRRAHRLLRARRLLKREGVLQAADFDENGLIKSLPNTPWQLRAAALDRKLTPL<br>EWSAVLLHLIKHRGYLSQRKNEGETADKELGALLKGVANNAHALQTGDFRTPAELALNKFE<br>KESGHIRNQRGDYSHTFSRKDLQAELILLFEKQKEFGNPHVSGGLKEGIETLLMTQRPALSGD

**as9**	AVQKMLGHCTFEPAEPKAAKNTYTAERFIWLTKLNNLRILEQGSERPLTDTERATLMDEPYR<br>KSKLTYAQARKLLGLEDTAFFKGLRYGKDNAEASTLMEMKAYHAISRALEKEGLKDKKSPL<br>NLSSELQDEIGTAFSLFKTDEDITGRLKDRVQPEILEALLKHISFDKFVQISLKALRRIVPLMEQ<br>GKRYDEACAEIYGDHYGKKNTEEKIYLPPIPADEIRNPVVLRALSQARKVINGVVRRYGSPA<br>RIHIETAREVGKSFKDRKEIEKRQEENRKDREKAAAKFREYFPNFVGEPKSKDILKLRLYEQQ<br>HGKCLYSGKEINLVRLNEKGYVEIDHALPFSRTWDDSFNNKVLVLGSENQNKGNQTPYEYF<br>NGKDNSREWQEFKARVETSRFPRSKKQRILLQKFDEDGFKECNLNDTRYVNRFLCQFVADHI<br>LLTGKGKRRVFASNGQITNLLRGFWGLRKVRAENDRHHALDAVVVACSTVAMQQKITRFV<br>RYKEMNAFDGKTIDKETGKVLHQKTHFPQPWEFFAQEVMIRVFGKPDGKPEFEEADTPEKL<br>RTLLAEKLSSRPEAVHEYVTPLFVSRAPNRKMSGAHKDTLRSAKRFVKHNEKISVKRVWLTE<br>IKLADLENMVNYKNGREIELYEALKARLEAYGGNAKQAFDPKDNPFYKKGGQLVKAVRVE<br>KTQESGVLLNKKNAYTIADNGDMVRVDVFCKVDKKGKNQYFIVPIYAWQVAENILPDIDCK<br>GYRIDDSYTFCFSLHKYDLIAFQKDEKSKVEFAYYINCDSSNGRFYLAWHDKGSKEQQFRIST<br>QNLVLIQKYQVNELGKEIRPCRLKKRPPVR
**FnCas9**	MNFKILPIAIDLGVKNTGVFSAFYQKGTSLERLDNKNGKVYELSKDSYTLLMNNRTARRHQR<br>RGIDRKQLVKRLFKLIWTEQLNLEWDKDTQQAISFLFNRRGFSFITDGYSPEYLNIVPEQVKAI<br>LMDIFDDYNGEDDLDSYLKLATEQESKISEIYNKLMQKILEFKLMKLCTDIKDDKVSTKTLKE<br>ITSYEFELLADYLANYSESLKTQKFSYTDKQGNLKELSYYHHDKYNIQEFLKRHATINDRILD<br>TLLTDDLDIWNFNFEKFDFDKNEEKLQNQEDKDHIQAHLHHFVFAVNKIKSEMASGGRHRS<br>QYFQEITNVLDENNHQEGYLKNFCENLHNKKYSNLSVKNLVNLIGNLSNLELKPLRKYFND<br>KIHAKADHWDEQKFTETYCHWILGEWRVGVKDQDKKDGAKYSYKDLCNELKQKVTKAGL<br>VDFLLELDPCRTIPPYLDNNNRKPPKCQSLILNPKFLDNQYPNWQQYLQELKKLQSIQNYLDS<br>FETDLKVLKSSKDQPYFVEYKSSNQQIASGQRDYKDLDARILQFIFDRVKASDELLLNEIYFQ<br>AKKLKQKASSELEKLESSKKLDEVIANSQLSQILKSQHTNGIFEQGTFLHLVCKYYKQRQRA<br>RDSRLYIMPEYRYDKKLHKYNNTGRFDDDNQLLTYCNHKPRQKRYQLLNDLAGVLQVSPN

	FLKDKIGSDDDLFISKWLVEHIRGFKKACEDSLKIQKDNRGLLNHKINIARNTKGKCEKEIFN<br>LICKIEGSEDKKGNYKHGLAYELGVLLFGEPNEASKPEFDRKIKKFNSIYSFAQIQQIAFAERK<br>GNANTCAVCSADNAHRMQQIKITEPVEDNKDKIILSAKAQRLPAIPTRIVDGAVKKMATILA<br>KNIVDDNWQNIKQVLSAKHQLHIPIITESNAFEFEPALADVKGKSLKDRRKKALERISPENIFK<br>DKNNRIKEFAKGISAYSGANLTDGDFDGAKEELDHIIPRSHKKYGTLNDEANLICVTRGDNK<br>NKGNRIFCLRDLADNYKLKQFETTDDLEIEKKIADTIWDANKKDFKFGNYRSFINLTPQEQK<br>AFRHALFLADENPIKQAVIRAINNRNRTFVNGTQRYFAEVLANNIYLRAKKENLNTDKISFDY<br>FGIPTIGNGRGIAEIRQLYEKVDSDIQAYAKGDKPQASYSHLIDAMLAFCIAADEHRNDGSIGL<br>EIDKNYSLYPLDKNTGEVFTKDIFSQIKITDNEFSDKKLVRKKAIEGFNTHRQMTRDGIYAEN<br>YLPILIHKELNEVRKGYTWKNSEEIKIFKGKKYDIQQLNNLVYCLKFVDKPISIDIQISTLEELR<br>NILTTNNIAATAEYYYINLKTQKLHEYYIENYNTALGYKKYSKEMEFLRSLAYRSERVKIKSI<br>DDVKQVLDKDSNFIIGKITLPFKKEWQRLYREWQNTTIKDDYEFLKSFFNVKSITKLHKKVR<br>KDFSLPISTNEGKFLVKRKTWDNNFIYQILNDSDSRADGTKPFIPAFDISKNEIVEAIIDSFTSKN<br>IFWLPKNIELQKVDNKNIFAIDTSKWFEVETPSDLRDIGIATIQYKIDNNSRPKVRVKLDYVID<br>DDSKINYFMNHSLLKSRYPDKVLEILKQSTIIEFESSGFNKTIKEMLGMKLAGIYNETSNN
Bl<br>at<br>Ca<br>s9	MAYTMGIDVGIASCGWAIVDLERQRIIDIGVRTFEKAENPKNGEALAVPRREARSSRRRLRR<br>KKHRIERLKHMFVRNGLAVDIQHLEQTLRSQNEIDVWQLRVDGLDRMLTQKEWLRVLIHLA<br>QRRGFQSNRKTDGSSEDGQVLVNVTENDRLMEEKDYRTVAEMMVKDEKFSDHKRNKGN<br>YHGVVSRSSLLVEIHTLFETQRQHHNSLASKDFELEYVNIWSAQRPVATKDQIEKMIGTCTFL<br>PKEKRAPKASWHFQYFMLLQTINHIRITNVQGTRSLNKEEIEQVVNMALTKSKVSYHDTRKI<br>LDLSEEYQFVGLDYGKEDEKKKVESKETIIKLDDYHKLNKIFNEVELAKGETWEADDYDTV<br>AYALTFFKDDEDIRDYLQNKYKDSKNRLVKNLANKEYTNELIGKVSTLSFRKVGHLSLKAL<br>RKIIPFLEQGMTYDKACQAAGFDFQGISKKKRSVVLPVIDQISNPVVNRALTQTRKVINALIK<br>KYGSPETIHIETARELSKTFDERKNITKDYKENRDKNEHAKKHLSELGIINPTGLDIVKYKLW<br>CEQQGRCMYSNQPISFERLKESGYTEVDHIIPYSRSMNDSYNNRVLVMTRENREKGNQTPFE

	YMGNDTQRWYEFEQRVTTNPQIKKEKRQNLLLKGFTNRRELEMLERNLNDTRYITKYLSHFI<br>STNLEFSPSDKKKKVVNTSGRITSHLRSRWGLEKNRGQNDLHHAMDAIVIAVTSDSFIQQVT<br>NYYKRKERRELNGDDKFPLPWKFFREEVIARLSPNPKEQIEALPNHFYSEDELADLQPIFVSR<br>MPKRSITGEAHQAQFRRVVGKTKEGKNITAKKTALVDISYDKNGDFNMYGRETDPATYEAI<br>KERYLEFGGNVKKAFSTDLHKPKKDGTKGPLIKSVRIMENKTLVHPVNKGKGVVYNSSIVRT<br>DVFQRKEKYYLLPVYVTDVTKGKLPNKVIVAKKGYHDWIEVDDSFTFLFSLYPNDLIFIRQN<br>PKKKISLKKRIESHSISDSKEVQEIHAYYKGVDSSTAAIEFIIHDGSYYAKGVGVQNLDCFEKY<br>QVDILGNYFKVKGEKRLELETSDSNHKGKDVNSIKSTSR
SpCas9	MDKKYSIGLDIGTNSVGWAVITDEYKVPSKKFKVLGNTDRHSIKKNLIGALLFDSGETAEAT<br>RLKRTARRRYTRRKNRICYLQEIFSNEMAKVDDSFFHRLEESFLVEEDKKHERHPIFGNIVDE<br>VAYHEKYPTIYHLRKKLVDSTDKADLRLIYLALAHMIKFRGHFLIEGDLNPDNSDVDKLFIQL<br>VQTYNQLFEENPINASGVDAKAILSARLSKSRRLENLIAQLPGEKKNGLFGNLIALSLGLTPNF<br>KSNFDLAEDAKLQLSKDTYDDDLDNLLAQIGDQYADLFLAAKNLSDAILLSDILRVNTEITK<br>APLSASMIKRYDEHHQDLTLLKALVRQQLPEKYKEIFFDQSKNGYAGYIDGGASQEEFYKFI<br>KPILEKMDGTEELLVKLNREDLLRKQRTFDNGSIPHQIHLGELHAILRRQEDFYPFLKDNREKI<br>EKILTFRIPYYVGPLARGNSRFAWMTRKSEETITPWNFEEVVDKGASAQSFIERMTNFDKNLP<br>NEKVLPKHSLLYEYFTVYNELTKVKYVTEGMRKPAFLSGEQKKAIVDLLFKTNRKVTVKQL<br>KEDYFKKIECFDSVEISGVEDRFNASLGTYHDLLKIIKDKDFLDNEENEDILEDIVLTLTLFEDR<br>EMIEERLKTYAHLFDDKVMKQLKRRRYTGWGRLSRKLINGIRDKQSGKTILDFLKSDGFANR<br>NFMQLIHDDSLTFKEDIQKAQVSGQGDSLHEHIANLAGSPAIKKGILQTVKVVDELVKVMGR<br>HKPENIVIEMARENQTTQKGQKNSRERMKRIEEGIKELGSQILKEHPVENTQLQNEKLYLYYL<br>QNGRDMYVDQELDINRLSDYDVDHIVPQSFLKDDSIDNKVLTRSDKNRGKSDNVPSEEVVK<br>KMKNYWRQLLNAKLITQRKFDNLTKAERGGLSELDKAGFIKRQLVETRQITKHVAQILDSR<br>MNTKYDENDKLIREVKVITLKSKLVSDFRKDFQFYKVREINNYHHAHDAYLNAVVGTALIK<br>KYPKLESEFVYGDYKVYDVRKMIAKSEQEIGKATAKYFFYSNIMNFFKTEITLANGEIRKRPL

	IETNGETGEIVWDKGRDFATVRKVLSMPQVNIVKKTEVQTGGFSKESILPKRNSDKLIARKK<br>DWDPKKYGGFDSPTVAYSVLVVAKVEKGKSKKLKSVKELLGITIMERSSFEKNPIDFLEAKG<br>YKEVKKDLIIKLPKYSLFELENGRKRMLASAGELQKGNELALPSKYVNFLYLASHYEKLKGS<br>PEDNEQKQLFVEQHKHYLDEIIEQISEFSKRVILADANLDKVLSAYNKHRDKPIREQAENIIHL<br>FTLTNLGAPAAFKYFDTTIDRKRYTSTKEVLDATLIHQSITGLYETRIDLSQLGGD
FrCas9	MCTKESEKLNKNADYYIGLDMGTSSAGWAVSDSEYNLIRRKGKDLWGVRQFEEAKTAAER<br>RGFRVARRRKQRQQVRNRLLSEEFQNEITKIDSGFLKRMEDSRFVISDKRVPEKYTLFNDSGY<br>TDVEYYNQYPTIYHLRKALIESNERFDIRLVFLGIHSLFQHPGHFLDKGDVDTDNTGPEELIQF<br>LEDCMNEIQISIPLVSNQKVLTDILTDSRITRRDKEQQILEILQPNKESKKAVSQFVKVLTGQK<br>AKLGDLIMMEDKDTEEYKYSFSFREKTLEEILPDIEGVIDGLALEYIESIYSLYSWSLLNSYMK<br>DTLTGHYYSYLAEARVAAYDKHHSDLVKLKTLFREYIPEEYDNFFRKMEKANYSHYIGSTE<br>YDGEKRCRTAKAKQEDFYKSINKMLEKIPECSEKTEIQKEIIEGTFLLKQTGPQNGFVPNQLQ<br>LKELRKILQNASKHYPFLTEKDERDMTAIDRIEALFSFRIPYYIGPLKNTDNQGHGWAVRRDG<br>HEQIPVRPWNFEEIIDESASADLFIKNLVNSCTYLRTEKVLPKSSLLYQEFEVLNELNNLRING<br>MYPDEIQPGLKRMIFEQCFYSGKKVTGKKLQLFLRSVLTNSSTEEFVLTGIDKDFKSSLSSYK<br>KFCELFGVKTLNDTQKVMAEQIIEWSTVYGDSRKFLKRKLEDNYPELTDQQIRRIAGFKFSE<br>WGNLSRAFLEMEGYKDEAGNPVTIIRALRDTQKNLMQLLSNDSAFAKKLQELNDYVTRDIW<br>SIEPDDLDGMYLSAPVRRMIWQTFLILREVVDTIGYSPKKIFMEMARGEQEKKRTASRKKQLI<br>DLYKEAGMKNDELFGDLESLEEAQLRSKKLYLYFRQMGRDIYSGKLIDFMDVLHGNRYDID<br>HIHPQSKKKDDSLENNLVLTSKDFNNHIKQDVYPIPEQIQSRQKGFWAMLLKQGFMSQEKYN<br>RLMRTTPFTDEELAEFVNRQLVETRQGTKAIISLINQCFPDSEVVYVKAGNTSDFRQRFDIPKS<br>RDLNNYHHAVDAYLNIVVGNVYDTKFTKNPINFIKKMRKSGNLHSYSL<br>RRMYDFNVQRGDQTAWVAENDTTLKTVKKTAFKTSPMVTKRTYERKGGLADSVLIAAKK<br>AKPGVHLPVKTSDSRFANQVSTYGGYDNVKGSHFFLVEHQQKKKTIRSIENVPIHLKEKLKT<br>KEELEHYCAQVLGMVQPDVRLTRIPMYSLLLIDGYYYYLTGRTGGNLSLSNAVELCLPAKE

	QAHIRMISKIAGGRSTDALSAEAKDDFRKKNLRLYDELAEKHRSTIFSKRKNPIGPKLLKYREAFVKQTIENQCKVILQILKLTSTNCKTSADLKLIGGSGQEGVMSISKLLRAEKYAEFYLICQSPSGIYETRKNLLTI

Supplementary Table 2. The CRISPR arrays of FrCas9 system.

Spacer start position	Spacer length	Spacer sequences
21248	31	TCAGGGTGCCTTTGCATCCATAATTCGGGTG
21315	30	TGTCCACCTTGCCATTGGCAAGCATTTGGG
21381	30	AATGCTGTTTGCGTTGGTCCATACGTACGA
21447	30	TGTACCAGGCATCTGCCCCTGCCACCGCCA
21513	30	TCAATTCCTCCAGGTCTTTGTCTCGACTTT
21579	31	CGATGGATGATCTGAAAAGCTATATGCGTGC
21646	30	CTTGCGACTGCAACTGTACTTACATTCTCT
21712	30	TAAAATCTCCTTTATATAAAGGTGATTAGC
21778	30	CCGCTCCCACTTGCTCACGATGGACTGGTC
21844	30	CGCGCTTATGGTTTCGCTTGCGCCTGTACT
21910	30	GATGCTTATCTTCGAAGAATAAAGCAACCG
21976	29	TGATTTTCTTGGTCATTGTCTTTTCCTCC
22041	30	AACGATGATTGCGACCGCTTCTGCTATGCC
22107	31	TGCCCTTGCTACCCGTGCCCGACTCTCCGAG
22174	30	GACTCCAAGAGTGTCAAGGGCCTGGCGTTT
22240	30	GTCACCGACACCGGTCTGGACAGCTGAATA

22306	30	CGAAGACGACCTCGAAACAGGGCCCGGCTT
22372	30	AAAGGCATACCACTGGTCCGTGATAGGCAC
22438	32	CAGATGTGTCAAACGGTATTCAGGGTCTTTGA
22506	30	ATCTTGCTGACACCATTCAGCGACCAGCGG
22572	30	AAGTCGTGTCGTCCGCCCAGTCCATCCGGG
22638	30	GACAAGATTGCCTTTCCCGCAGCGGATGGT
22704	30	GTTCCTGTTCAAGAGTTATAAGGGCGGACA
22770	30	GCATCGCGATGAACCACTTGACCACGTCCT
22836	31	ACAAGGCTCCGCGCTGGATTGTCTGGAATCT
22903	30	ATTCCACTGCATGGTCTGTGTCTTCGCACT
22969	31	ACGATGGTCCGAAGCGCTGGCTCAACTGCGG
23036	30	GATGACCTGTCTAAAATCCTTCCTCAGTCT
23102	31	CTCCGCTGCCTGCAATACCAGCTATCACAAT
23169	30	TGATGAATCCGCGCATCATGTAGGTCGCAT
23235	31	CCTGTTCTGTCATTACAGTAGAAGGGGAATT
**DR start position**	**DR length**	**Direct repeat sequences**
21212	36	GTTTGAGTGTCTTGTTAATTCGGAAGTATTTCAAAC
21279	36	GTTTGAGTGTCTTGTTAATTCGGAAGTATTTCAAAC
21345	36	GTTTGAGTGTCTTGTTAATTCGGAAGTATTTCAAAC
21411	36	GTTTGAGTGTCTTGTTAATTCGGAAGTATTTCAAAC
21477	36	GTTTGAGTGTCTTGTTAATTCGGAAGTATTTCAAAC
21543	36	GTTTGAGTGTCTTGTTAATTCGGAAGTATTTCAAAC

21610	36	GTTTGAGTGTCTTGTTAATTCGGAAGTATTTCAAAC
21676	36	GTTTGAGTGTCTTGTTAATTCGGAAGTATTTCAAAC
21742	36	GTTTGAGTGTCTTGTTAATTCGGAAGTATTTCAAAC
21808	36	GTTTGAGTGTCTTGTTAATTCGGAAGTATTTCAAAC
21874	36	GTTTGAGTGTCTTGTTAATTCGGAAGTATTTCAAAC
21940	36	GTTTGAGTGTCTTGTTAATTCGGAAGTATTTCAAAC
22005	36	GTTTGAGTGTCTTGTTAATTCGGAAGTATTTCAAAC
22071	36	GTTTGAGTGTCTTGTTAATTCGGAAGTATTTCAAAC
22138	36	GTTTGAGTGTCTTGTTAATTCGGAAGTATTTCAAAC
22204	36	GTTTGAGTGTCTTGTTAATTCGGAAGTATTTCAAAC
22270	36	GTTTGAGTGTCTTGTTAATTCGGAAGTATTTCAAAC
22336	36	GTTTGAGTGTCTTGTTAATTCGGAAGTATTTCAAAC
22402	36	GTTTGAGTGTCTTGTTAATTCGGAAGTATTTCAAAC
22470	36	GTTTGAGTGTCTTGTTAATTCGGAAGTATTTCAAAC
22536	36	GTTTGAGTGTCTTGTTAATTCGGAAGTATTTCAAAC
22602	36	GTTTGAGTGTCTTGTTAATTCGGAAGTATTTCAAAC
22668	36	GTTTGAGTGTCTTGTTAATTCGGAAGTATTTCAAAC
22734	36	GTTTGAGTGTCTTGTTAATTCGGAAGTATTTCAAAC
22800	36	GTTTGAGTGTCTTGTTAATTCGGAAGTATTTCAAAC
22867	36	GTTTGAGTGTCTTGTTAATTCAGAAGTATTTCAAAC
22933	36	GTTTGAGTGTCTTGTTAATTCGGAAGTATTTCAAAC
23000	36	TTTTGAGTGTCTTGTTAATTCGGAAGTATCTCAAAC
23066	36	GTTTGAGTGTCTTGTTAATTCGGAAGTATTCCAAAC

23133	36	GTTTGAGTGTCTTGTTAATTCGGAAGTATTTCAAAC
23199	36	GTTTGAGTGTCTTGTTAATTCGGAAGTAAGCTCAAC
23266	36	GTTTGAGTGTCTTGTTAATTCGGGAGTAGCTCTCTC

Supplementary Table 3. The tracrRNA sequences of FrCas9

tracrRNA	AATTAACAAGATGAGTTCAAATCAGGCTCCTAGAGAGATCCGAACTTACCTTCATGGCGGGCATTGTGCCC

Supplementary Table 4. The domain locations of FrCas9.

Start	End	Domain
1	60	RuvC-I
54	750	REC
750	799	RuvC-II
804	959	HNH
963	1096	RuvC-III
1093	1372	PI

Supplementary Table 5. The targets used in FrCas9 prokaryotic and eukaryotic validation.

Target name	PAM	Guide sequence (5' to 3')	Assay
PAM library	XXXXXX	TGACTTTAAAAGTATTCG CCAT	Plasmid depletion assay
PAM library	GGTA	TGACTTTAAAAGTATTCG CCAT	Plasmid interference assay
PAM library	XXXXXX	TGACTTTAAAAGTATTCG CCAT	Puromycin depletion assay
RNF2	CGTA	CTTCATTGAGATTAGATTT CAA	Eukaryotic validation
GRIN2B	AGTA	AAGATTTAGGGTCATATG TGGA	Eukaryotic validation
DYRK1A	TGTA	AGAATTAAATCATCTTTT AAAA	Eukaryotic validation
RNF2	CATA	ACCTGAGGTGTTCGTTGT AACT	Eukaryotic validation
GRIN2B	GATA	TCGTGACTTCCCTAAAAT GACT	Eukaryotic validation
DYRK1A	TATA	TTAATACACTATGTTCTTC CAG	Eukaryotic validation
RNF2	ACTA	ACAACGAACACCTCAGGT AATG	Eukaryotic validation

GRIN2B	GCTA	TGGCTCTAACATTACTTCA GCT	Eukaryotic validation
DYRK1A	ACTA	CTATGATATGTGTTCAACT TGC	Eukaryotic validation
RNF2	GTTA	CATGGGAACTCAGTTTAT ATGA	Eukaryotic validation
GRIN2B	CTTA	ACTTCCACATATGACCCT AAAT	Eukaryotic validation
DYRK1A	TTTA	ACTGGATTGAGGTCTGGT ACAT	Eukaryotic validation
RNF2	CGTA	CTTCATTGAGATTAGATTT CAA	Eukaryotic validation
GRIN2B	AGTA	AAGATTTAGGGTCATATG TGGA	Eukaryotic validation
DYRK1A	TGTA	AGAATTAAATCATCTTTT AAAA	Eukaryotic validation
RNF2	CATA	ACCTGAGGTGTTCGTTGT AACT	Eukaryotic validation
RNF2-T6	GGTA	ATGAGTTACAACGAACAC CTCA	Scaffold architecture
HEK293 SITE xT2	GGTA	TGAGCTAACTGTGACAGC ATGT	Guide sequence length

DNMT1-T3	AGTA	GTGTCACGCCACTTGACA GGCG	Guide sequence length
RNF2-T6	GGTA	ATGAGTTACAACGAACAC CTCA	Guide sequence length
ANAPC15	CGTA	TGGTCAACTGCATGGTCA TTAT	Guide sequence length
DNMT1-T2	CGTA	ACTAGATCTACCAGTATC AACA	Guide sequence length
GRIN2B-T6	TGTA	TGGAGAAGGGGCTGCCTA AGGA	Guide sequence length
RNF2-T3	CGTA	ATCAGTGGTGGCATTAGA CTCT	Guide sequence length
RUNX1-T7	TGTA	TTCTCTAAGTTGGTGACCT AAT	Guide sequence length
Mismatch target	GGTA	ACGCTCGCAGATCATCAT AGCG	Point mutation sgRNAs assay

Supplementary Table 6. The GUIDE-seq sgRNA

Site	PAM	sgRNA sequence	Cas9
DYRK1A-T2	GGTA	TGTTACCACTGGATTGAGGTCT	FrCas9
EMX1-T2	GGTA	GGTCATTGTCATGTCCAGTTGT	FrCas9
FANCF-T3	GGTA	CGGGGTCCCAGGTGCTGACGTA	FrCas9
FANCF-T4	GGTA	AAGCGCCGATGGATGTGGCGCA	FrCas9
GRIN2B-T3	GGTA	TGGCCATGCGACCCTCTTCATA	FrCas9
GRIN2B-T8	GGTA	AGAAAGCATAGGTTTCAAAACA	FrCas9
GRIN2B-T9	GGTA	AAAAAGGAAGGAGTTCTTTGTA	FrCas9
GRIN2B-T14	GGTA	AATTGGAGGGCACATTTTTGCC	FrCas9
HEK293 SITE xT2	GGTA	TGAGCTAACTGTGACAGCATGT	FrCas9
RNF2-T6	GGTA	ATGAGTTACAACGAACACCTCA	FrCas9
RUNX1-T3	GGTA	AACCTCACCCTTGGAGAGTTCA	FrCas9
RUNX1-T5	GGTA	TTAGTATAAGCGCTCAATCAAT	FrCas9
DYRK1A-T	TGG	GTTACCACTGGATTGAGGTC	SpCas9
EMX1-T2	TGG	GTCATTGTCATGTCCAGTTG	SpCas9
FANCF-T3	AGG	GGGGTCCCAGGTGCTGACGT	SpCas9
FANCF-T4	AGG	AGCGCCGATGGATGTGGCGC	SpCas9
GRIN2B-T3	AGG	GGCCATGCGACCCTCTTCAT	SpCas9
GRIN2B-T8	AGG	GAAAGCATAGGTTTCAAAAC	SpCas9
GRIN2B-T9	AGG	AAAAGGAAGGAGTTCTTTGT	SpCas9
GRIN2B-T14	CGG	ATTGGAGGGCACATTTTTGC	SpCas9
HEK293 SITE xT2	TGG	GAGCTAACTGTGACAGCATG	SpCas9

RNF2-T6	AGG	TGAGTTACAACGAACACCTC	SpCas9
RUNX1-T3	AGG	ACCTCACCCTTGGAGAGTTC	SpCas9
RUNX1-T5	TGG	TAGTATAAGCGCTCAATCAA	SpCas9

Supplementary Table 7. The list of primers used in this study.

Primer name	Sequence	Assay
Library-insert-F	GTACGACTGACTCGGACATA	Construction of plasmid library
Library-insert-R	CTACAATCGGCTCGATCGAT	Construction of plasmid library
Library-vector-F	TATGTCCGAGTCAGTCGTACCGT ATGGGGCTGACTTCAGG	Construction of plasmid library
Library-vector-R	ATCGATCGAGCCGATTGTAGAC CGGGTCGAATTTGCTTTC	Construction of plasmid library
NGS1-F	ACACTCTTTCCCTACACGACGCT CTTCCGATCTGTCTACAATCGGC TCGATCGA	Deep sequencing of Plasmid depletion assay
NGS1-R	GTGACTGGAGTTCAGACGTGTG CTCTTCCGATCTGCGCAGACCAA AACGATCTC	Deep sequencing of Plasmid depletion assay
GGTA-F	ACTTTAAAAGTATTCGCCATGGT AGCCCGGGTACCGAGCTCGAAT	Construction of target plasmid
GGTA-R	CTACCATGGCGAATACTTTTAAA GTGATCCTCTAGAGTCGACCTGC AGGC	Construction of target plasmid
Puromycin-insert-F	ATGAACCATGGACATGACTT	Construction of plasmid library

Puromycin-insert-R	AATGCAGCTGGCACGAATTC	Construction of plasmid library
Puromycin-vector-F	GAATTCGTGCCAGCTGCATTACCGAGTACAAGCCCACGGT	Construction of plasmid library
Puromycin- vector -R	AAGTCATGTCCATGGTTCATCATGGAAGGTCGTCTCCTTG	Construction of plasmid library
NGS2-F	ACACTCTTTCCCTACACGACGCTCTTCCGATCTCCACAAGGAGACGACCTTCC	Deep sequencing of puromycin depletion assay
NGS2-R	GTGACTGGAGTTCAGACGTGTGCTCTTCCGATCTGTGACCCGCTCGATGTGG	Deep sequencing of puromycin depletion assay
RNF2-T6-F	AGTGTCTGGTGAGGGCCTAT	dsODN breakpoint-PCR
ODN-F	TTGAGTTGTCATATGTTAATAACGGT	dsODN breakpoint-PCR
ODN-R	ACCGTTATTAACATATGACAACTCAA	dsODN breakpoint-PCR
gg-RNF2-T6-F1	ACACTCTTTCCCTACACGACGCTCTTCCGATCTTATTTCCAGCAATGTCTCAGGCT	Deep sequencing of scaffold architecture
gg-RNF2-T6-R1	GTGACTGGAGTTCAGACGTGTGCTCTTCCGATCTGTTTTCATGTTCTAAAAATGTATCCCA	Deep sequencing of scaffold architecture

Target-insert-F	ACGCTCGCAGATCATCATAGCGGGTAGAGACAAATGGCTCTAGAGGT	Construction of plasmid library
Target-insert-R	CTACCCGCTATGATGATCTGCGAGCGTAAAAAAAGGGCACAATGCC	Construction of plasmid library
Mismatch-F	ACACTCTTTCCCTACACGACGCTCTTCCGATCTGGGTTTATATATCTTGTGGAAAGGA	Deep sequencing of point mutation sgRNAs assay
Mismatch-R	GTGACTGGAGTTCAGACGTGTGCTCTTCCGATCTACCCTAACTGACACACAT	Deep sequencing of point mutation sgRNAs assay
HEK293 SITE2-T2-F	ACGGAATGAATGGATTCCTTGGA	Guide sequence length
HEK293 SITE2-T2-R	TCCAGCCCCATCTGTCAAAC	Guide sequence length
DNMT1-T3-F	GGGACCGTTTGAGGAGTGTT	Guide sequence length
DNMT1-T3-R	GGGAGGGCAGAACTAGTCCT	Guide sequence length
RNF2-T6-F	AGTGTCTGGTGAGGGCCTAT	Guide sequence length
RNF2-T6-R	ACCACTGTTCACCCAGTACC	Guide sequence length
ANAPC15-F	CGACCAGGAGTGCCAGATAA	Guide sequence length
ANAPC15-R	CGTCTCCTTGCCCCTTCATT	Guide sequence length
DNMT1-T2-F	TTCGTGGCCCCATCTTTCTC	Guide sequence length

DNMT1-T2-R	CCAAGGCCACAAACACCATG	Guide sequence length
GRIN2B-T6-F	TAGACCCAGCACCCCATGTA	Guide sequence length
GRIN2B-T6-R	AGAGTGCCTTGTCCAGAGAA	Guide sequence length
RNF2-T3-F	TGAGAATGCACCATGATTCCA	Guide sequence length
RNF2-T3-R	CCCAGGGACCAGTTTCATCC	Guide sequence length
RUNX1-T7-F	ACCTCTGCTATCCCGAGGG	Guide sequence length
RUNX1-T7-R	GTGTGTGTCCCAGGTTTCCA	Guide sequence length
DYRK1A-T2-F	TCCAGTTTCTCCCGATCCTCT	GUIDE-seq dsODN breakpoint-PCR
EMX1-T2-F	TCCTGGGAATCTCTTGTGCT	GUIDE-seq dsODN breakpoint-PCR
FANCF-T3-F	CAGCATGTGCACCGCAGA	GUIDE-seq dsODN breakpoint-PCR
FANCF-T4-F	CAGCATGTGCACCGCAGA	GUIDE-seq dsODN breakpoint-PCR
GRIN2B-T3-F	AACCTTTGGGCTTCAGGAGG	GUIDE-seq dsODN breakpoint-PCR
GRIN2B-T8-F	ATCTGGGGGAAGGCTCTGAA	GUIDE-seq dsODN breakpoint-PCR
GRIN2B-T9-F	TGTATTGGCATGCTGGTGTT	GUIDE-seq dsODN breakpoint-PCR
GRIN2B-T14-F	CTCATATGTGCCTGAGCCGT	GUIDE-seq dsODN breakpoint-PCR

HEK293 SITE2-T2-F	AAGGAAAAGCTCTGTCATCCA	GUIDE-seq dsODN breakpoint-PCR
RNF2-T6-F	AGTGTCTGGTGAGGGCCTAT	GUIDE-seq dsODN breakpoint-PCR
RUNX1-T3-F	CATTCAAGGAATGCTGAAGGC	GUIDE-seq dsODN breakpoint-PCR
RUNX1-T5-F	GACGTCACAACTTAAAAACGTGC	GUIDE-seq dsODN breakpoint-PCR
ABCA1-qPCR-F	GAGCCGGTCATCAATCTCAT	qPCR primers of CRISPRi/CRISPRa
ABCA1-qPCR-R	CCAACCTCATCAGGAAGCAT	qPCR primers of CRISPRi/CRISPRa
UCP3-qPCR-F	TCCGTCAAGCAGGTGTACAC	qPCR primers of CRISPRi
UCP3-qPCR-R	CTGGCCTGAAATCGGACCTT	qPCR primers of CRISPRi
RANKL-qPCR-F	CCCTTGCCCACCTCATGATT	qPCR primers of CRISPRi
RANKL-qPCR-R	AGCCTAGGTTCCGTAGAGCA	qPCR primers of CRISPRi
SOD1-qPCR-F	GAAGGTGTGGGGAAGCATTAAAG	qPCR primers of CRISPRa
SOD1-qPCR-R	AGAGGATTAAAGTGAGGACCTGC	qPCR primers of CRISPRa
GH1-qPCR-F	AGCAACGTCTATGACCTCCTAA	qPCR primers of CRISPRa
GH1-qPCR-R	GCAGGAATGTCTCGACCTTGT	qPCR primers of CRISPRa
MBL2-qPCR-F	GTGATGGCACCAAGGGAGAA	qPCR primers of CRISPRa

MBL2-qPCR-R	TCCAGGGTCTCCTTTTTGGC	qPCR primers of CRISPRa

Raw gel images of Supplementary Fig. 5a

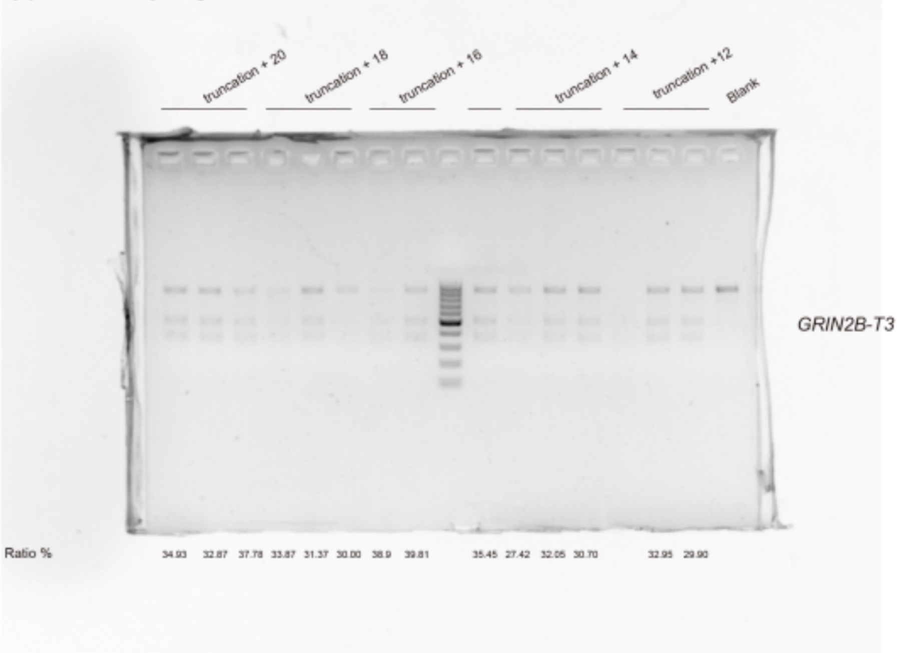

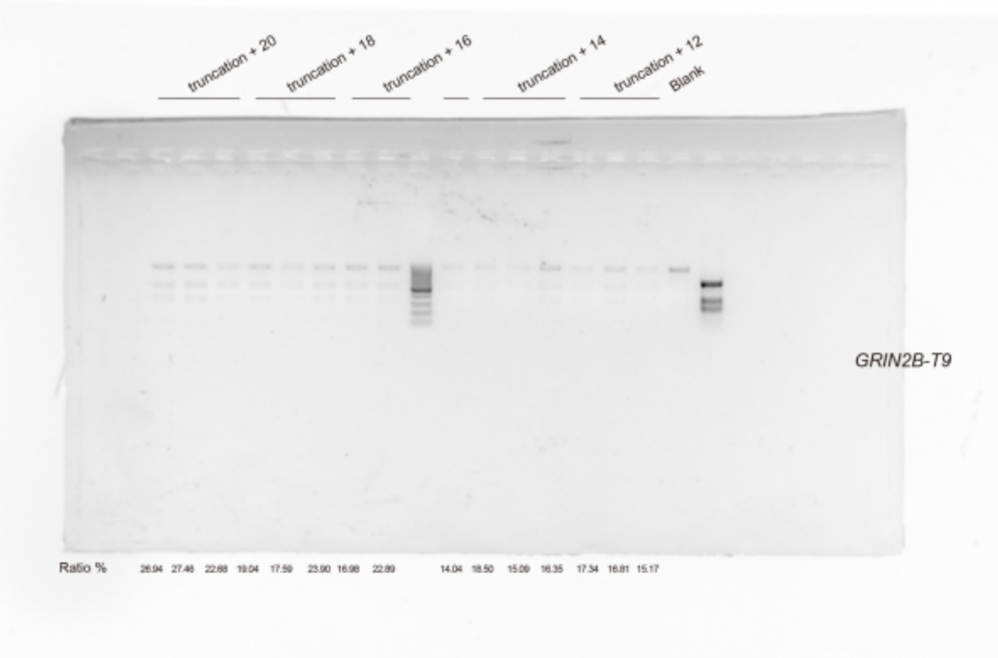

Raw gel images of Supplementary Fig. 7

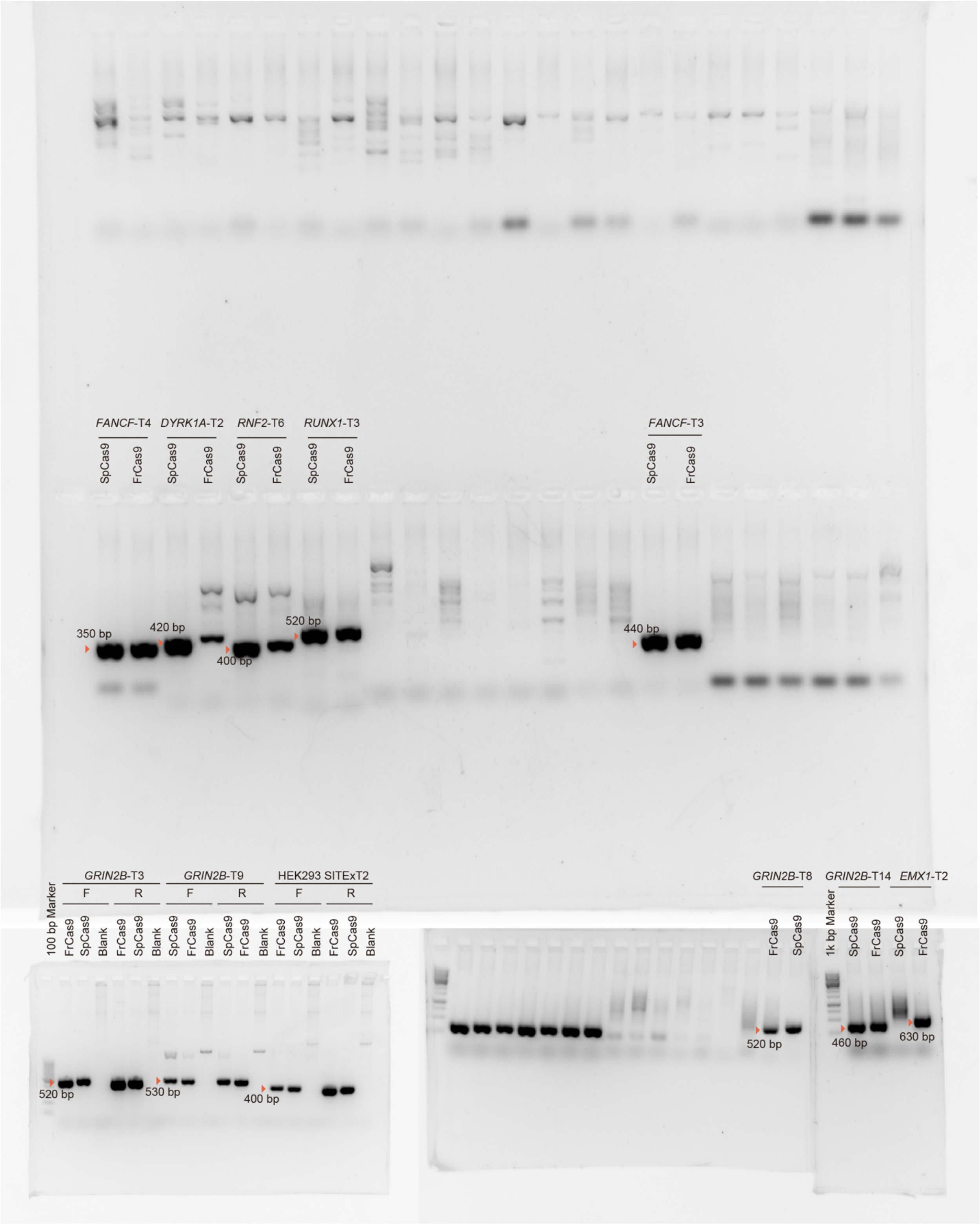

Raw gel images of Supplementary Fig. 9a

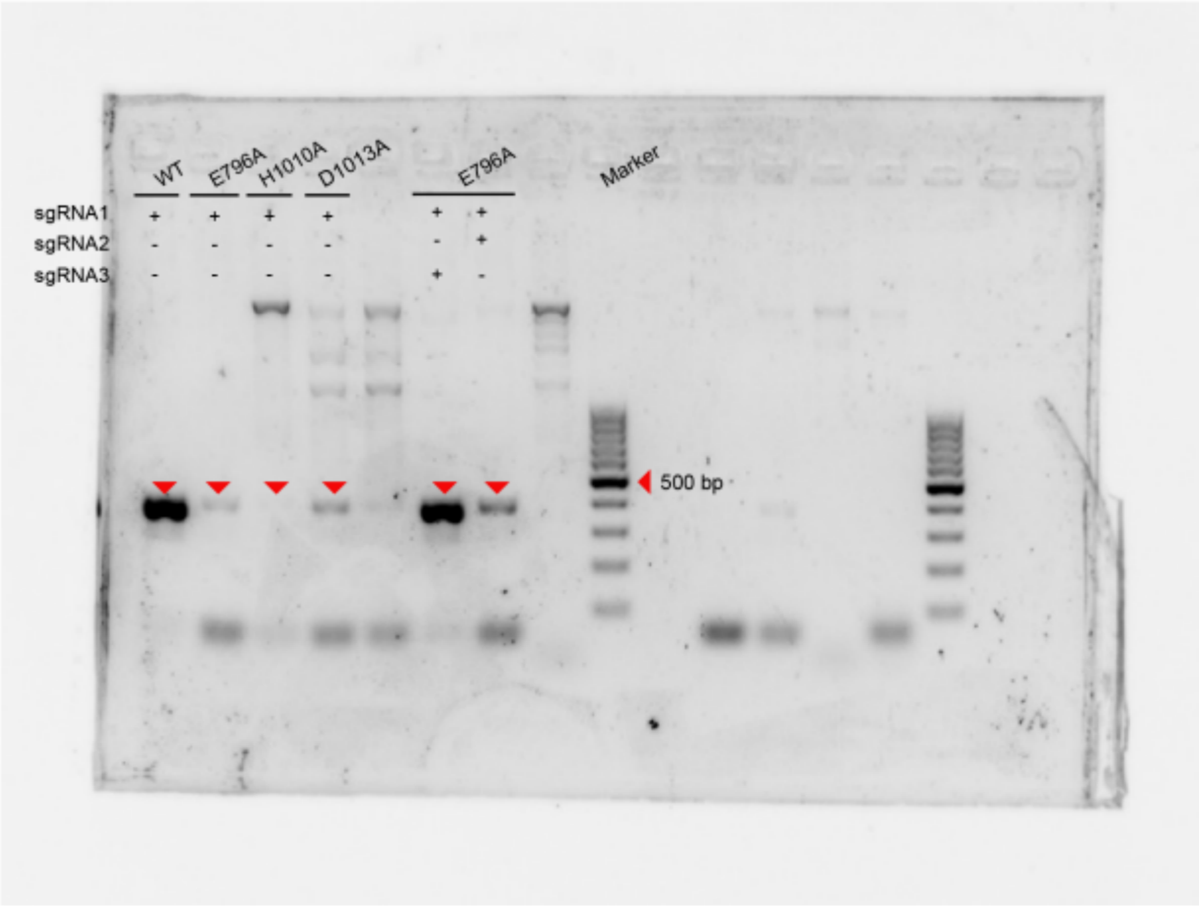

Supplement: Supplementary file 1 — Supplementary information [file 41467_2022_29089_MOESM1_ESM.pdf]
