# Supplementary material for: FrCas9 is a CRISPR/Cas9 system with high editing efficiency and fidelity
Source: Nat Commun. 2022 Mar 17;13:1425. doi: 10.1038/s41467-022-29089-8 (PMC8931148; doi:10.1038/s41467-022-29089-8)
Supplement: Supplementary file 2 — Reporting Summary [file 41467_2022_29089_MOESM2_ESM.pdf]

Corresponding author(s): Zheng Hu

Last updated by author(s): Feb 23, 2022

Reporting Summary

Nature Portfolio wishes to improve the reproducibility of the work that we publish. This form provides structure for consistency and transparency in reporting. For further information on Nature Portfolio policies, see our Editorial Policies and the Editorial Policy Checklist.

Statistics

For all statistical analyses, confirm that the following items are present in the figure legend, table legend, main text, or Methods section.

n/a	Confirmed	
☐	☒	The exact sample size (*n*) for each experimental group/condition, given as a discrete number and unit of measurement
☐	☒	A statement on whether measurements were taken from distinct samples or whether the same sample was measured repeatedly
☐	☒	The statistical test(s) used AND whether they are one- or two-sided<br>*Only common tests should be described solely by name; describe more complex techniques in the Methods section.*
☒	☐	A description of all covariates tested
☒	☐	A description of any assumptions or corrections, such as tests of normality and adjustment for multiple comparisons
☐	☒	A full description of the statistical parameters including central tendency (e.g. means) or other basic estimates (e.g. regression coefficient) AND variation (e.g. standard deviation) or associated estimates of uncertainty (e.g. confidence intervals)
☐	☒	For null hypothesis testing, the test statistic (e.g. *F*, *t*, *r*) with confidence intervals, effect sizes, degrees of freedom and *P* value noted<br>*Give P values as exact values whenever suitable.*
☒	☐	For Bayesian analysis, information on the choice of priors and Markov chain Monte Carlo settings
☒	☐	For hierarchical and complex designs, identification of the appropriate level for tests and full reporting of outcomes
☒	☐	Estimates of effect sizes (e.g. Cohen's *d*, Pearson's *r*), indicating how they were calculated

Our web collection on statistics for biologists contains articles on many of the points above.

Software and code

Policy information about availability of computer code

Data collection	No software was used.
Data analysis	We annotated the genome with standalone CRISPRCasFinder software (version: 2.0.2). Then we predicted the secondary structures of crRNA and tracrRNA using NUPCAK software (http://www.nupack.org). For FrCas9 protein, we used the HHpred software (https://toolkit.tuebingen.mpg.de/tools/hhpred) to predict the functional domains, and based on the prediction results we extracted FrCas9 PAM Interaction (PI) domain. The phylogenetic trees of Cas9 orthologs and PI domains were constructed using FastTree software (version: 2.1.11) with the "-wag -gamma" parameter after alignment by MAFFT software (version: 7.490). The PAM sequences of each Cas9 ortholog in phylogenetic trees were made by Weblogo3 (http://weblogo.threeplusone.com/) using matrixs. The HT-PAMDA data was analyzed by the scripts in Github (https://github.com/kleinstiverlab/HT-PAMDA).The small RNA-seq data was visualized by Sushi R package (version: 3.14).The GUIDE-seq sequencing data was demultiplexed by bcl2fastq software (version: 1.8.4).The custom codes was deposited in Github (https://github.com/Freya-Cui-2020/FrCas9)

For manuscripts utilizing custom algorithms or software that are central to the research but not yet described in published literature, software must be made available to editors and reviewers. We strongly encourage code deposition in a community repository (e.g. GitHub). See the Nature Portfolio guidelines for submitting code & software for further information.

Data

Policy information about availability of data

All manuscripts must include a data availability statement. This statement should provide the following information, where applicable:

- Accession codes, unique identifiers, or web links for publicly available datasets
- A description of any restrictions on data availability
- For clinical datasets or third party data, please ensure that the statement adheres to our policy

Source data are provided with this paper. The genome of Faecalibaculum rodentium strain NYU-BL-K8 is available in NCBI database under accession code NZ_MPJZ01000004.1. The sequencing data generated in this study have been deposited in the NCBI Sequence Read Archive (SRA) database under the accession code PRJNA766437.

Field-specific reporting

Please select the one below that is the best fit for your research. If you are not sure, read the appropriate sections before making your selection.

[x] Life sciences [ ] Behavioural & social sciences [ ] Ecological, evolutionary & environmental sciences

For a reference copy of the document with all sections, see nature.com/documents/nr-reporting-summary-flat.pdf

Life sciences study design

All studies must disclose on these points even when the disclosure is negative.

Sample size	No statistical method was used to determine sample sizes and at least three repeats were used for important experiments to draw reliable conclusions.
Data exclusions	No data were excluded from the analyses
Replication	Important experiments were repeated three times and all attempts at replication were successful.
Randomization	Samples (cells) were allocated into experimental groups randomly.
Blinding	Samples (cells) were allocated into experimental groups blindly and more than one investigators observed analysis results independently.

Reporting for specific materials, systems and methods

We require information from authors about some types of materials, experimental systems and methods used in many studies. Here, indicate whether each material, system or method listed is relevant to your study. If you are not sure if a list item applies to your research, read the appropriate section before selecting a response.

Materials & experimental systems

n/a	Involved in the study	
[x]	[ ]	Antibodies
[ ]	[x]	Eukaryotic cell lines
[x]	[ ]	Palaeontology and archaeology
[x]	[ ]	Animals and other organisms
[x]	[ ]	Human research participants
[x]	[ ]	Clinical data
[x]	[ ]	Dual use research of concern

Methods

n/a	Involved in the study	
[x]	[ ]	ChIP-seq
[ ]	[x]	Flow cytometry
[x]	[ ]	MRI-based neuroimaging

Eukaryotic cell lines

Policy information about cell lines

Cell line source(s)	HeLa (CCL2, ATCC), U2OS (HTB-96, ATCC) and HEK293T (CRL3216, ATCC) cell lines were purchased from ATCC.
Authentication	Short tandem repeat (STR) testing was performed to confirm cell identity.
Mycoplasma contamination	All cell lines were cultured at 37 °C with 5% CO2 and tested negative for mycoplasma contamination using MycoAlert Mycoplasma Detection kit.
Commonly misidentified lines (See ICLAC register)	No commonly misidentified cell lines were used in the study.

Flow Cytometry

Plots

Confirm that:

- [x] The axis labels state the marker and fluorochrome used (e.g. CD4-FITC).
- [x] The axis scales are clearly visible. Include numbers along axes only for bottom left plot of group (a 'group' is an analysis of identical markers).
- [x] All plots are contour plots with outliers or pseudocolor plots.
- [x] A numerical value for number of cells or percentage (with statistics) is provided.

Methodology

Sample preparation	HeLa cells were seeded into 12-well plates and transfected with 2.5 μg FrCas9-gRNA plasmid using SE Cell Line 4D-Nucleofector™ X Kit. At 48 h post transfection, cells were collected and stained using an Annexin V-APC/PI Apoptosis detection kit (KGA1030, Key GEN BioTECH, China).
Instrument	flow cytometry (Gallios, Beckman Coulter, USA) and Kaluza Analysis software (Beckman Coulter, USA).
Software	Data and statistical analyses were done in FlowJo 10, unless otherwise stated. The statistical details of the experiments are provided in the respective figure legends.
Cell population abundance	Cell death was scored by quantifying the population of Annexin V-FITC-positive cells for 10,000 events
Gating strategy	Depending on fluorescence intensity of Annexin V and PI, the populations can be distinguished into double negative (healthy) cells, Annexin-V positive(early apoptotic cells) and double positive (late apoptotic and necroptotic) cells.

- [x] Tick this box to confirm that a figure exemplifying the gating strategy is provided in the Supplementary Information.
